# Few-Shot Cross-Domain Fault Diagnosis via Wavelet Convolution Embedding and BDC-Based Metric Meta-Learning

**DOI:** 10.3390/s26134276

**Published:** 2026-07-05

**Authors:** Zaiyou Xu, Jiale Kai, Jun Wang

**Affiliations:** School of Rail Transportation, Soochow University, Suzhou 215131, China; 18896919620@163.com (Z.X.); kerwin020812@gmail.com (J.K.)

**Keywords:** meta-learning, few-shot fault diagnosis, cross-domain generalization, Brownian distance covariance, wavelet convolution, prototypical network

## Abstract

Few-shot cross-domain bearing fault diagnosis is challenging because labeled fault samples are limited and signals collected by vibration sensors under different operating conditions often show significant distribution shifts. To improve bearing fault identification under limited-sample and cross-condition scenarios, this paper proposes a wavelet convolution (WC) and Brownian distance covariance (BDC)-based metric meta-learning framework, termed WCBDC. In this framework, the WC is inserted into the feature extraction process to capture multiscale time–frequency information from vibration signals. The BDC is then applied to model nonlinear inter-channel statistical dependencies and improve the discriminability of fault embeddings. The obtained feature embeddings are further organized within a prototypical-network-based classifier, in which category prototypes are estimated from support samples and query instances are assigned by prototype-distance comparison. The proposed method is evaluated on the Paderborn University (PU) and Beijing Jiaotong University (BJTU) bearing datasets under both 5-way 5-shot and 5-way 1-shot scenarios. On the PU dataset, WCBDC reaches average accuracies of 92.19% and 84.13%, while the corresponding results on the BJTU dataset are 77.24% and 62.57%. These results exceed those of representative meta-learning baselines, demonstrating that WCBDC provides improved diagnostic performance for sensor-based bearing fault recognition when labeled samples are scarce and operating conditions vary.

## 1. Introduction

Industrial fault diagnosis plays an important role in modern manufacturing systems by ensuring the reliability, safety, and long-term stable operation of critical machinery components such as rolling bearings [1]. Accurate diagnosis can help prevent unexpected breakdowns and cascading failures while reducing downtime and maintenance costs, which is essential for efficient and sustainable intelligent manufacturing [2]. With the development of Industry 4.0 and condition-based maintenance, intelligent fault diagnosis (IFD) has become a key technique for condition monitoring and predictive maintenance [3]. A typical IFD pipeline includes fault detection, isolation, and identification based on vibration signals collected by machinery sensors. Among these steps, fault identification is particularly critical because it directly affects diagnostic accuracy and subsequent maintenance decisions [4]. However, accurate fault identification remains challenging, as industrial machinery often operates under complex working conditions and generates non-stationary vibration signals [5].

In conventional fault diagnosis frameworks, measured signals are generally first transformed into manually defined representations before classification is performed [6]. Fault-discriminative information is usually characterized from multiple signal perspectives, including temporal, spectral, and time–frequency representations, where the short-time Fourier transform and wavelet transform (WT) are frequently adopted as typical analysis tools [7]. Based on these extracted descriptors, traditional pattern recognition algorithms, such as support vector machines, k-nearest neighbors, and artificial neural networks, are then employed to distinguish different fault categories [8]. Although these methods can achieve satisfactory performance under laboratory conditions, they strongly depend on expert knowledge and are sensitive to environmental and operating-condition variations [9]. In engineering practice, rotating machinery rarely works under a strictly stationary operating condition; instead, its vibration responses are continuously modulated by load, speed, and environmental variations, which makes fault-related patterns more difficult to distinguish [10]. Consequently, handcrafted descriptors tend to be condition-dependent and may suffer from degraded transferability across domains, ultimately compromising diagnostic robustness [11]. The increasing complexity of industrial systems has therefore motivated the development of adaptive data-driven methods with automatic feature learning and better cross-domain generalization [12].

Instead of relying on manually designed descriptors, recent intelligent diagnosis studies increasingly employ deep neural networks to learn fault representations from raw sensor data [13]. CNNs, autoencoders, and recurrent neural networks are commonly used for this purpose, as they can extract hierarchical and class-discriminative information from vibration signals with reduced dependence on handcrafted features [14]. In bearing fault identification, adversarial training has been incorporated into CNNs to enhance robustness against input perturbations [15]. Convolutional autoencoders have also been adopted to suppress noise in vibration measurements while constructing compact diagnostic representations for rotating machinery [16]. Beyond these models, graph convolutional networks coupled with long short-term memory networks have been explored to describe temporal dependencies and relational information embedded in vibration sequences [17]. However, the effectiveness of deep learning-based diagnosis is often contingent on large-scale labeled datasets and relatively invariant operating conditions, both of which are difficult to guarantee in real industrial scenarios [18].

In industrial practice, collecting sufficient labeled fault samples is prohibitively expensive and time-consuming, as failures are rare and system downtime must be minimized [19]. Furthermore, data distributions across operating conditions often vary due to fluctuating speed, load, and environmental noise, leading to severe domain shifts [20]. As a result, deep learning models trained in one domain tend to experience significant performance degradation when applied to new or unseen conditions [21]. These constraints have encouraged many studies on fault diagnosis under limited labeled samples and cross-domain scenarios [22]. Few-shot learning frameworks enable rapid adaptation using only a few labeled samples from novel fault categories [23], while domain adaptation and transfer-learning approaches have been proposed to mitigate distribution discrepancies by aligning feature representations across source and target domains [24]. Digital twin-enabled fault diagnosis has also been explored by combining virtual data and real monitoring signals to improve bearing fault diagnosis under limited real fault data [25]. Digital twin-guided physical–virtual denoising methods further show advantages in enhancing weak fault features for early bearing fault detection [26]. Despite these efforts, achieving robust generalizable performance under both few-shot and cross-domain constraints remains challenging [27].

Meta-learning, often described as “learning to learn”, provides an effective way to address few-shot diagnosis problems by learning transferable knowledge from multiple related tasks [28]. Among different meta-learning strategies, metric-based methods have been widely studied because of their simple classification mechanism and low computational cost [29]. These methods usually construct an embedding space in which samples from the same class are close to each other, while samples from different classes are separated. Some studies further improve generalization by introducing adversarial or contrastive learning to reduce distribution discrepancies [30]. Recent studies have shown that metric-based few-shot learning can achieve competitive performance in data-scarce scenarios by measuring the distance between query samples and support prototypes [31]. In industrial fault diagnosis, such methods have also been applied to few-shot cross-domain diagnosis tasks for bearings and gearboxes [32]. Although these approaches have shown good adaptability, two limitations remain. First, conventional convolutional encoders may not sufficiently capture the multiscale time–frequency characteristics of vibration signals, which are important for identifying subtle fault patterns under varying operating conditions [33]. Second, existing methods may not fully capture nonlinear feature relationships in complex mechanical systems, reducing the discriminability and robustness of the learned representations [34].

To address these limitations, this paper proposes a meta-learning-based fault diagnosis framework that integrates wavelet convolution (WC) and Brownian distance covariance (BDC) into a prototypical network. The proposed framework is termed WCBDC. The WC module integrates wavelet decomposition into convolutional feature extraction, allowing the model to capture multiscale time–frequency information from vibration signals. The BDC module is applied to measure nonlinear statistical dependencies between feature channels, thereby improving the discriminability and stability of the embedding space. By coupling wavelet-enhanced feature learning with BDC-based embedding representation, the proposed WCBDC model is designed to improve few-shot fault diagnosis across varying operating domains. The main contributions of this study are summarized as follows:

1. A unified few-shot diagnostic framework is developed by incorporating wavelet convolution and Brownian distance covariance into a metric-learning paradigm, allowing the model to adapt efficiently to previously unseen operating conditions;
2. The wavelet convolution module is designed to embed wavelet decomposition and reconstruction into CNN-based representation learning, so that multiscale time–frequency characteristics of vibration signals can be captured in an end-to-end manner;
3. The Brownian distance covariance module is employed to characterize both linear and nonlinear dependencies among feature channels, thereby enhancing the separability and robustness of fault embeddings under domain variations.

The rest of this paper is arranged as follows. Section 2 formulates the diagnostic task and presents the metric-based meta-learning framework. Section 3 details the proposed WCBDC architecture, with emphasis on the wavelet convolution and Brownian distance covariance modules. Section 4 reports the experimental setup, comparison results, and ablation analysis. Section 5 gives the conclusions.

## 2. Theoretical Background

### 2.1. Metric-Based Meta-Learning

**Figure 1:**
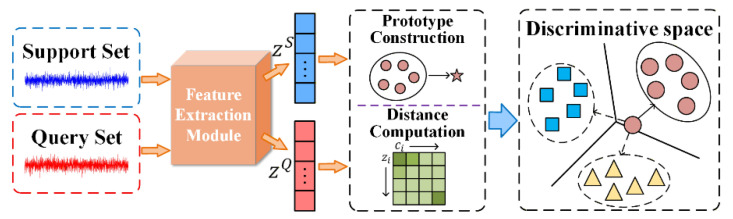
Metric-based meta-learning framework.

Figure 1 portrays the integral layout of a metric-governed meta-learning paradigm. In this construct, support and query exemplars are transformed by the encoder f(⋅) into embeddings, whereby the raw measurements are projected onto a class-separable representational manifold: samples sharing identical labels are agglomerated, while those from disparate categories are dispersed [35]. Metric-driven approaches adjudicate classes through inter-sample separations in the embedding space rather than through wholesale parameter readjustment, which diminishes computational expenditure and restrains overfitting tendencies [36].

Cross-domain fault diagnosis is recast into a collection of few-shot learning tasks, where each task $T_{j}$ consists of a support set $S={\left\{(x_{i}^{S},y_{i}^{S})\right\}}_{i=1}^{n_{s}}$ and a query set $Q={\left\{(x_{i}^{Q},y_{i}^{Q})\right\}}_{i=1}^{n_{Q}}$, with $n_{S}\ll n_{Q}$. The support ensemble offers a meager allotment of labeled exemplars to facilitate model accommodation, while the query ensemble functions as the benchmark for evaluative assessment.

During meta-training, tasks are sampled from a task distribution $D^{r}$, and the optimization objective is formulated as

(1) $$\omega^{*}=\underset{\omega }{\mathrm{argmin}}E_{T_{j}\sim D^{r}}[L(T_{j};\omega )]$$

where ω denotes the learnable parameters of the model, $\omega^{*}$ is the optimized solution obtained after meta-training, $E_{T_{j}\sim D^{r}}[\cdot ]$ represents the expectation over sampled tasks, L(⋅) is the objective function designed to compel within-class tightness and between-class divergence, thereby amplifying the separability and distinctiveness of the learned embeddings.

Given the optimized parameters $\omega^{*}$, the embedding representations of support and query samples are first obtained as

(2) $$z_{i}^{S}=f_{\omega^{*}}\left(x_{i}^{S}\right),\;\;z_{i}^{Q}=f_{\omega^{*}}(x_{i}^{Q})$$

where $z_{i}^{S}\;$and $z_{i}^{Q}\;$represent feature embeddings of support and query samples, respectively, and class prototypes are derived by averaging support embeddings over each class:

(3) $$p_{c}=\frac{1}{\left|S_{c}\right|}\sum_{(x_{i}^{S},y_{i}^{c})\in S_{c}}z_{i}^{S}$$

wherein $p_{c}$ denotes the prototypical locus of category c, while $S_{c}$ refers to the corresponding support ensemble comprising samples affiliated with category c.

To perform classification, similarity between query embeddings and class prototypes is measured by the squared Euclidean distance $d(p_{c},z_{i}^{Q})=∥p_{c}-z_{i}^{Q}{∥}_{2}^{2}$, and the corresponding class probability is computed using a softmax over negative distances:

(4) $$P(y=c|z_{i}^{Q})=e^{-d\left(p_{c},z_{i}^{Q}\right)}/\sum_{c^{\prime }=1}^{N^{\prime }}e^{-d\left(p_{c^{\prime }},z_{i}^{Q}\right)}$$

The above formulation provides a unified metric-based learning framework for few-shot classification, where feature representation, prototype construction, and distance-based similarity measurement are jointly integrated within a consistent embedding space.

### 2.2. Brownian Distance Covariance

**Figure 2:**
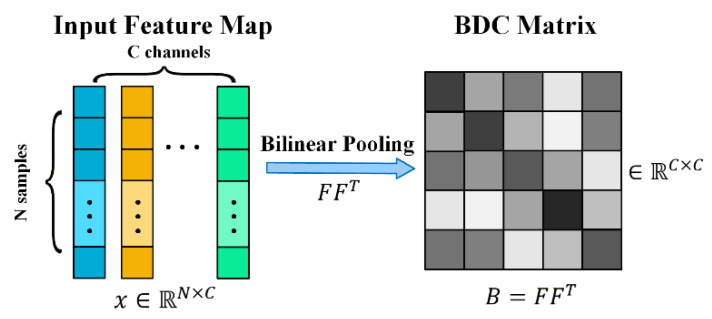
Brownian distance covariance computation.

Brownian distance covariance (BDC) measures statistical dependence between feature channels, capturing both linear and nonlinear correlations [37]. As shown in Figure 2, BDC transforms convolutional feature maps into embeddings that encode inter-channel dependencies. Its formulation rests on the conjoint characteristic function of the duplet of random vectors $X\in R^{p}$ and $Y\in R^{q}$:

(5) $$\phi_{XY}\left(t,s\right)=E\left[e^{i\left(t^{T}X+s^{T}Y\right)}\right]=\int_{R^{p}}\int_{R^{q}}e^{i\left(t^{T}X+s^{T}Y\right)}f_{XY}\left(x,y\right)dxdy$$

where $t\in R^{p}$ and $s\in R^{q}$, $f_{XY}(x,y)$ denotes the joint density of X and Y, and i satisfies $i^{2}=-1$. The corresponding marginal characteristic functions are denoted as $\phi_{X}(t)$ and $\phi_{Y}(s)$, respectively.

Grounded on these definitions, BDC is expressed as the weighted $L_{2}$ distance separating the joint characteristic function from the product of the marginal characteristic functions:

(6) $$\rho^{2}\left(X,Y\right)=\int_{R^{p}}\int_{R^{q}}\frac{{\left|\phi_{XY}\left(t,s\right)-\phi_{X}\left(t\right)\phi_{Y}\left(s\right)\right|}^{2}}{c_{p}c_{q}{\left\|t\right\|}^{1+p}{\left\|s\right\|}^{1+q}}dt\;ds$$

where $c_{p}$ and $c_{q}$ are positive dimension-dependent constants. Under this definition, independence between X and Y is equivalent to ρ(X,Y)=0, which enables BDC to characterize statistical dependence effectively.

For practical applications in deep learning, the continuous formulation of BDC is difficult to compute directly. Therefore, a sample-based approximation is commonly adopted, where statistical dependence is estimated using pairwise distances between feature representations. Specifically, given feature vectors extracted from a neural network, BDC characterizes inter-sample dependencies by constructing a Euclidean distance matrix. A subsequent centering operation is applied to remove mean effects and obtain a normalized representation of the dependency structure. This procedure results in a compact statistical descriptor that captures the intrinsic relationships among feature components, making BDC effective for modeling complex dependencies in high-dimensional feature spaces.

## 3. Proposed WCBDC Model

### 3.1. Overall Architecture

**Figure 3:**
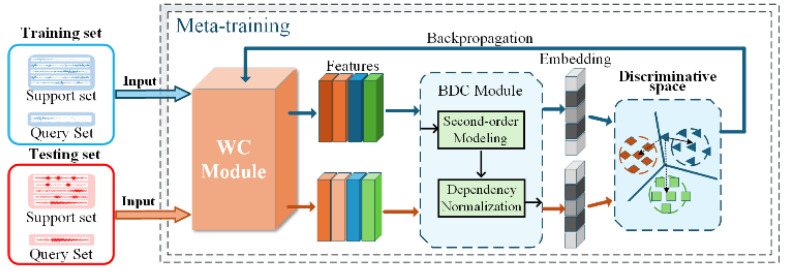
Global schematic of the envisaged WCBDC construct.

Figure 3 presents the structure of WCBDC, which follows a metric-based meta-learning paradigm augmented with WC and BDC modules. The overall pipeline is structured into three phases: data construction, meta-training, and meta-testing. Through the incorporation of WC and BDC modules, the framework enables robust fault classification under few-shot and cross-domain conditions. Further details are elaborated below.

In this architecture, the WC module is used to replace the first conventional convolutional layer of the CNN encoder, rather than serving as an additional independent branch. It first extracts multiscale time–frequency features from the input vibration signal. The subsequent CNN blocks further transform these features into high-level feature maps, which are then fed into the BDC module. The BDC module generates a vectorized embedding by modeling inter-channel statistical dependencies. Finally, the obtained embeddings are used in the prototypical classifier, where support embeddings are averaged to form class prototypes and query embeddings are classified according to their Euclidean distances to these prototypes.

#### 3.1.1. Data Construction

Initially, the raw vibration flux is dissected into segments of fixed span, each segment functioning as a discrete sample. Segment length is dictated by the interplay of sampling cadence and the temporal scales of fault-indicative patterns. The derived samples are then bifurcated into training and testing assemblages for meta-training and meta-testing operations, respectively.

Embedded in the meta-learning framework, the model is iteratively trained and assessed across a series of meta-tasks. Every meta-task comprises multiple categorical classes, each containing a support set and a query set. As an illustration, when each category is endowed with five support exemplars and five query exemplars, the arrangement embodies a prototypical N-way K-shot schema.

#### 3.1.2. Meta-Training Process

The meta-training stage constitutes the core of the WCBDC model, and its functionality can be divided into two parts: embedding construction and metric-based classification.

Firstly, one-dimensional (1-D) vibration signals are mapped into a discriminative embedding space through the embedding function $f_{\theta }(\cdot )$. A given input signal $x\in R^{L}$ is transformed into an embedding vector $z=f_{\theta }(x)$, where $z\in R^{D}$. The embedding function designed in this study includes the WC module and BDC module, which is formulated as

(7) $$z=f_{\theta }\left(x\right)=BDC(CNN({WC}_{\theta }\left(x\right)))$$

The WC module extracts local time–frequency representations, while the BDC module enhances inter-sample correlation discriminability, providing stable embeddings for subsequent prototype-based metric classification.

Based on the obtained embeddings z, metric-based classification is performed following the prototypical learning framework described in Section 2, where similarity is measured in the embedding space to obtain the predictive distribution P(y∣z).

The model is trained using a cross-entropy objective computed on the query set Q:

(8) $$L=-\sum_{\left(x_{i},y_{i}\right)\in Q}\log P\left(y=c_{i}|z_{i}\right)$$

Finally, during inference, the predicted label is determined as

(9) $$\hat{y}=\underset{c}{\mathrm{argmax}}P(y=c|z)$$

### 3.2. WC Module

The WC module combines the advantages of wavelet transform (WT) and CNNs to extract multiscale time–frequency features from vibration signals. The WT is particularly suitable for analyzing non-stationary signals, capturing local variations and specific frequency components of rotating-machinery faults. Compared with standard convolution layers that are more responsive to high-frequency components, WC guides the network to better capture low-frequency information by cascading wavelet decomposition and reconstruction.

#### 3.2.1. Wavelet Transform Theory

In this study, the Daubechies wavelet with vanishing moment 10 (db10) is employed owing to the effective time–frequency localization and suitability for vibration signal analysis in rotating machinery fault diagnosis.

For computational implementation, the WT is realized via 1-D convolutions using a pair of fixed filters: a low-pass filter (LPF) $f_{L}$ and high-pass filter (HPF) $f_{H}$ derived from db10. Since db10 has a filter length of 20, the filters are expressed as

(10) $$f_{L}=\left[h_{1},h_{2},\ldots ,h_{20}\right],f_{H}=[g_{1},g_{2},\ldots ,g_{20}]$$

where $h_{i}$ and $g_{i}$ denote the scaling and wavelet coefficients of the db10 filter bank, which are used to generate the approximation branch and the detail branch, respectively. These coefficients are fixed by the db10 construction and remain unchanged during training, thereby ensuring a stable and physically interpretable wavelet decomposition process.

Specifically, convolution is applied to decompose the input signal x into low-frequency and high-frequency components:

(11) $$X_{L}=x*f_{L},\;\;X_{H}=x*f_{H}$$

where ∗ denotes 1-D convolution, and the stride is set to 2, corresponding to a single-level wavelet decomposition. Here, $X_{L}$ captures the low-frequency trend information, while $X_{H}$ represents high-frequency transient details. The boundary-handling properties of the wavelet kernels enhance the ability of the network to capture long-term dependencies, while simultaneously reducing the influence of irrelevant information.

#### 3.2.2. Designed WC Module

**Figure 4:**
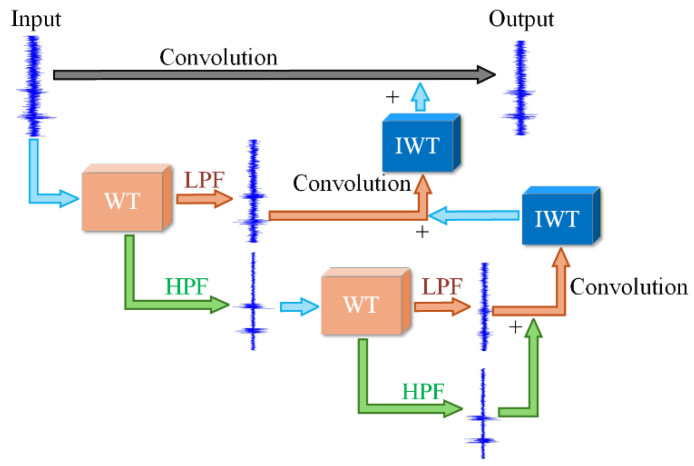
Architecture of the Designed WC Module.

The designed WC module is illustrated in Figure 4. In addition to the WT branch, a parallel 1-D convolutional branch is introduced to capture complementary local features. Specifically, a convolution function Conv() with kernel size 3×1 is adopted in this branch. The output of the WC module is obtained through feature fusion, formulated as

(12) $$F_{WC}=F_{WT}^{1}+Conv(x)$$

where $F_{WT}^{1}$ is the output of the WT branch, in which the low- and high-frequency components are separately processed via depth-wise separable convolutions and reconstructed using the inverse wavelet transform (IWT):

(13) $$F_{WT}^{1}=IWT(Conv(X_{L}^{\left(1\right)})+F_{WT}^{2})$$

where $X_{L}^{\left(1\right)}$ is the low-frequency components of the original sample x, and $F_{WT}^{2}$ is obtained via:

(14) $$F_{WT}^{2}=IWT(Conv(X_{L}^{\left(2\right)})+X_{H}^{\left(2\right)})$$

where $X_{L}^{\left(2\right)}$ and $X_{H}^{\left(2\right)}$ are the low- and high-frequency components of $X_{H}^{\left(1\right)}$, respectively, and $X_{H}^{\left(1\right)}$ is the high-frequency component of the original sample x.

This hierarchical fusion integrates multi-scale information, improving the richness of extracted representations.

#### 3.2.3. BDC Module

After feature extraction by the WC module, the resulting feature map is $F_{WC}\in R^{B\times d\times l}$, where B is the batch size, d is the number of feature channels, and l is the feature length. For each sample, the feature map is written as $F\in R^{d\times l}$. The BDC module then captures inter-channel dependencies by constructing a distance-aware embedding from F. In this way, the theoretical BDC formulation is implemented in the network by estimating channel-wise statistical relationships from the learned finite-dimensional feature map. First, a Gram matrix G is computed as

(15) $$G=FF^{T},\;G\in R^{d\times d}$$

where $G\;consists\;of\;G_{ij}$ that denotes the inner-product similarity between channel i and j. The diagonal elements $G_{ii}$ represent channel-wise self-activation strengths, while the off-diagonal elements characterize inter-channel dependencies.

Based on the Gram matrix, the pairwise squared Euclidean distance matrix $\tilde{A}$ $\in R^{d\times d}$ between channels is computed as

(16) $${\tilde{A}}_{ij}=G_{ii}+G_{jj}-2G_{ij}$$

which encodes the structural dissimilarity between channel representations derived from inner-product similarities.

A learnable temperature parameter t is introduced to rescale the distance distribution:

(17) $$\tilde{A}=e^{t}\cdot \tilde{A},\;t\in R$$

which adjusts the global distance scale to better control the distribution of pairwise relationships.

To enhance numerical stability and introduce nonlinearity, an element-wise transformation is applied as

(18) $${\hat{A}}_{ij}=\sqrt{{\tilde{A}}_{ij}+\epsilon },\;\epsilon >0$$

where $\hat{A}$ denotes the element-wise square-root transformed distance matrix and ϵ is used to prevent numerical instability. To eliminate global bias while preserving relative channel dependencies, a double-centering operation is performed as

(19) $$A=\hat{A}-\frac{1}{d}\hat{A}1-\frac{1}{d}1\hat{A}+\frac{1}{d^{2}}1\hat{A}1$$

where $A\in R^{d\times d}$ is the centered dependency matrix, and $1\in R^{d\times d}$ is an all-ones matrix. This operation removes global bias while preserving relative inter-channel dependencies, thereby improving the stability of the learned representation under distribution variations.

Finally, the upper-triangular elements of A are extracted to form the final BDC embedding:

(20) $$F_{final}={vec}_{△}\left(A\right),\;F_{final}\in R^{\frac{d(d+1)}{2}}$$

where ${vec}_{△}(\cdot )$ extracts the upper-triangular elements of a symmetric matrix and $F_{final}$ denotes the final embedding obtained from BDC output.

Overall, the BDC module maps the WC-extracted features into a compact embedding space by modeling inter-channel statistical dependencies. The resulting BDC embedding is then used for prototype construction and query classification in the prototypical network.

## 4. Experimental Results and Discussion

### 4.1. Dataset Description

#### 4.1.1. PU Bearing Dataset

**Figure 5:**
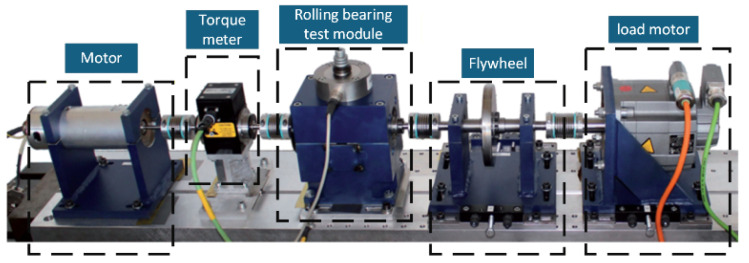
Test rig of the PU bearing dataset.

The PU bearing corpus [38] was procured at Paderborn University in Germany, with the corresponding test apparatus portrayed in Figure 5. This experimental assemblage integrates an electric motor, a torque-sensing shaft, a rolling-bearing inspection module, a flywheel, and a loading motor, while 6203-type bearings serve as the examined elements.

The PU dataset contains 32 fault categories: 12 artificially damaged bearings, 14 accelerated life-induced faults, and 6 healthy bearings categorized based on operation duration. The dataset provides a wide range of operating conditions. For each class, samples are available in the following four cases—N15_M07_F10, N09_M07_F10, N15_M01_F10, and N15_M07_F04—corresponding to different combinations of rotational speed (RS), load torque, and radial force (F_r), as listed in Table 1. For instance, in N15_M07_F10, “N15” indicates a rotational speed setting of 1500 rpm, “M07” indicates a load torque of 0.7 Nm, while “F10” corresponds to a radial force of 1000 N.

In this study, all 32 official fault categories are used for experiments. Variations in fault severity, manufacturing method, and arrangement are treated as distinct classes to enable fine-grained classification granularity. Cross-domain experiments are conducted across the four operating conditions to ensure diversity and experimental comprehensiveness.

#### 4.1.2. BJTU Bearing Dataset

**Figure 6:**
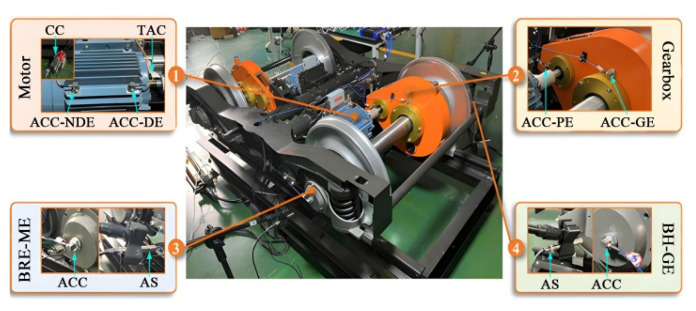
Metro bogie simulation test bench of Beijing Jiaotong University.

The BJTU axle-box bearing dataset [39], now openly accessible, stems from the National Key Laboratory for Advanced Rail Autonomous Operation of Beijing Jiaotong University. As delineated in Figure 6, this corpus was harvested from a simplified 1:2-scaled experimental apparatus abstracted from a real metro bogie system. Its power-transmission route is constituted by a motor, a reduction gearbox, and an axle box. Propulsion is furnished by a three-phase asynchronous motor, with its rotational velocity modulated via a variable-frequency drive. External excitation is exerted through a hydraulic loading mechanism. The axle-box bearings are specified as HRB 352213, and the bearing under inspection resides in the left axle box. Vibration traces were captured from the x-direction of a triaxial accelerometer affixed to the axle-box end cover, under a sampling frequency of 64 kHz. Altogether, the corpus contains eleven fault-state categories, including one intact state, four isolated faults, and six compound faults. Four operating regimes were arranged for cross-domain evaluation, each regime corresponding to a distinct concatenation of motor speed and lateral load, as enumerated in Table 2.

In both datasets, each category contains 500 samples per operating condition, each consisting of 512 vibration data points. No sample is repeated during acquisition. During meta-training, a 5-way 5-shot task configuration is used. Meta-testing is conducted under both 5-way 5-shot and 5-way 1-shot regimes, with the class cardinality in both support and query sets preserved as identical for each task. For cross-domain evaluation, 100 samples of each category are tested for each operating condition. In the PU experiments, 32 fault categories were used to construct 128 tasks for 5-way 5-shot diagnosis and 640 tasks for 5-way 1-shot diagnosis. The BJTU dataset contained 11 fault categories, yielding 44 and 220 tasks for the two diagnosis settings, respectively. Each meta-test was run ten times, and the reported accuracy was obtained by averaging the repeated results.

### 4.2. Few-Shot Cross-Domain Diagnosis Experiments

In the few-shot cross-domain diagnosis experiments, each episode is constructed as a 5-way *K*-shot task. Five fault categories are randomly selected from the corresponding operating domain, and K labeled samples are chosen from each category as the support set. In this study, K=5 is used for meta-training, while K∈{1,5} is used for meta-testing. Meanwhile, Q query samples are selected from each category, with Q=5 in all experiments. The support and query samples do not overlap. For a cross-domain task denoted as a→b, training episodes are constructed from Domain a, whereas testing episodes are constructed from Domain b, ensuring strict separation between source and target operating conditions.

#### 4.2.1. Compared Methods

In comparative experiments, besides the proposed WCBDC model, four additional models are considered for benchmarking, as detailed below.

Prototypical Network (PN): PN is a canonical metric-governed meta-learning scheme in which each category is epitomized by the averaged embedding of its support exemplars. Classification is subsequently adjudicated through Euclidean separation, thereby endowing the method with comparatively economical computation.

Model-Agnostic Meta-Learning (MAML): MAML is a paradigmatic optimization-driven meta-learning strategy that seeks an initialization of model parameters amenable to swift accommodation when confronted with novel tasks.

Relation Network (RN): It further introduces a learnable relation module to capture nonlinear similarity relationships between feature embeddings, improving discriminative performance.

Enhanced Transformer with Asymmetric Loss Function (ETALF): It combines a transformer architecture with an asymmetric loss function to enhance robustness against noisy labels and improve cross-domain generalization ability in few-shot fault diagnosis scenarios.

#### 4.2.2. Backbone CNN Architecture

The backbone CNN adopted in this work consists of four cascaded convolutional blocks, where each block integrates a convolutional layer, batch normalization, ReLU nonlinearity, and a 2 × 2 max-pooling operation. The convolutional layers utilize 3 × 3 kernels with a stride set to one.

In Block 1, the network commences with 1 input channel and expands to 32 feature channels. After batch normalization, activation, and pooling, the feature map size is reduced from 512 to 256.

Block 2 increases the number of channels to 256 while reducing the spatial dimension to 128 after pooling. Similarly, Block 3 decreases channels to 64 and reduces the feature map to 64, and Block 4 maintains 64 channels while reducing the feature map to 32.

This hierarchical design allows the network to efficiently extract multilevel features, gradually increasing the channel dimension while reducing the spatial size. For fair comparison, the same CNN backbone is used as the encoder for all the compared methods.

#### 4.2.3. Experiment Settings for Ablation Studies

For ablation studies, BDC and WC are individually removed from the baseline network separately, directly flattening the features. The BDC model employs the BDC module only, without WC, whereas the WC model uses only WC and performs direct feature flattening without BDC integration.

#### 4.2.4. Training Configuration

All experiments are performed in a uniform environment: Ubuntu 22.04 (Canonical Ltd., London, UK), PyTorch 2.10.0+cu130 (PyTorch Foundation, Linux Foundation, San Francisco, CA, USA), GPU: NVIDIA GeForce RTX 3070 Ti (NVIDIA Corporation, Santa Clara, CA, USA). Training consists of 200 epochs, with 50 tasks per epoch. The initial learning rate (lr) is set to 0.001 and is decayed stepwise to 0.0001 and 0.00001 at 70% and 90% of the total training epochs, respectively. This stepwise decay strategy ensures stable convergence while mitigating the impact of learning-rate variations on model performance.

**Figure 7:**
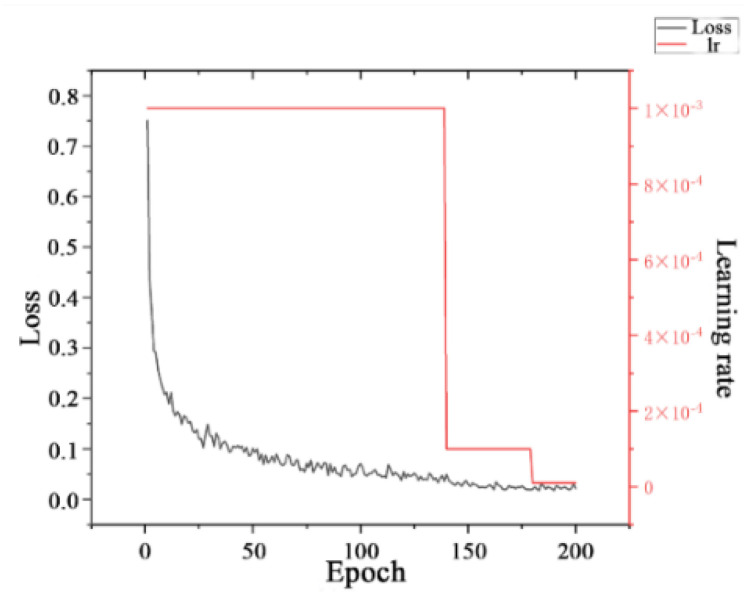
Training dynamics of loss with stepwise learning-rate decay.

**Figure 8:**
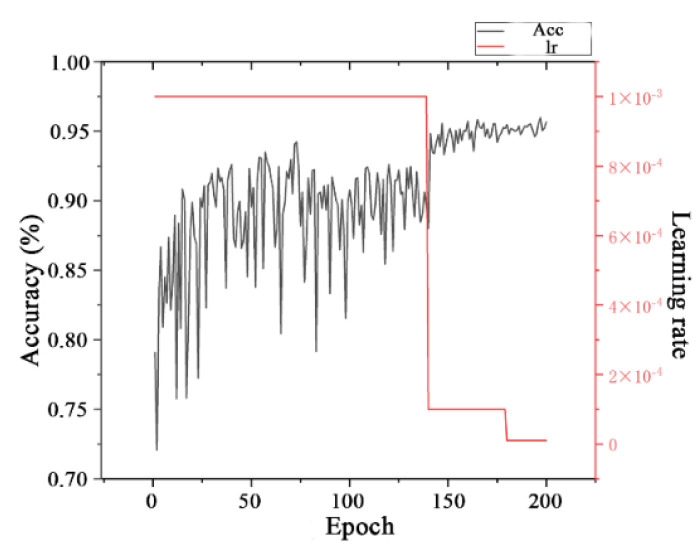
Training dynamics of validation accuracy with stepwise learning-rate decay.

As shown in Figure 7, using WCBDC as an example, the training loss decreases rapidly at the initial stage. When the learning rate decays in epochs 140 and 180, the loss stabilizes. Similarly, the validation accuracy exhibits large fluctuations at early stages and gradually reaches its peak and stabilizes as the learning rate steps down, as presented in Figure 8. These outcomes substantiate that the adopted training strategy effectively allows the model to achieve optimal performance, ensuring the reliability of the experimental results.

### 4.3. Experimental Results

#### 4.3.1. Comparative Experiments

For the PU dataset, Table 3 and Table 4, respectively, enumerate the few-shot cross-domain diagnostic results obtained under the 5-way 5-shot and 5-way 1-shot meta-testing configurations. The best mean accuracy for each task is accentuated in boldface. It can be discerned from the results that the proposed WCBDC model invariably secures the highest accuracy among all task-wise comparisons, attaining average accuracies of 92.19% in the 5-way 5-shot regime and 84.13% in the 5-way 1-shot regime. For typical 5-way 5-shot transfer tasks, such as 0→1, 0→2, and 1→2, WCBDC records accuracies of 93.54%, 93.77%, and 97.17%, respectively, thereby exceeding all selected reference methods. ETALF ranks immediately behind WCBDC, while PN, MAML, and RN display relatively diminished diagnostic efficacy. Collectively, these findings attest to the superior few-shot cross-domain fault-identification competence of the WCBDC method on the PU dataset.

**Figure 9:**
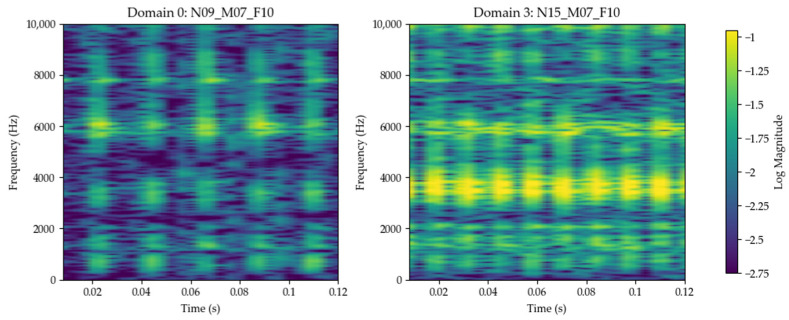
Time–frequency comparison between PU Domain 0 and Domain 3 under the same fault category.

Although WCBDC achieves the best average performance on the PU dataset, Table 3 and Table 4 show that several tasks with Domain 0 as the target domain, such as 1→0, 2→0, and 3→0, still obtain relatively lower accuracies. To further illustrate this phenomenon, Figure 9 compares N09_M07_F10 (Domain 0) and N15_M07_F10 (Domain 3) under the same representative fault category and signal length. These two conditions are selected because they have the same load torque and radial force but different rotational speeds, allowing the effect of speed variation to be highlighted. The different time–frequency energy distributions suggest that speed variation may change fault-related frequency components, making feature alignment between source and target domains more difficult.

The comparative results on the BJTU dataset are catalogued in Table 5 and Table 6. WCBDC generally achieves better overall performance than the competing methods, yielding average accuracies of 77.24% for the 5-way 5-shot tasks and 62.57% for the 5-way 1-shot tasks. ETALF assumes the runner-up position, reaching 70.33% in the 5-way 5-shot configuration, while PN, MAML, and RN tend to generate inferior and more erratic diagnostic outcomes, especially under transfer tasks accompanied by substantial condition-induced variability. The comparative evidence suggests that embedding formation governed by BDC, together with WC-based feature elicitation, materially enhances diagnostic performance. Across the PU and BJTU datasets, the proposed WCBDC model exhibits marked generalizability and resilience in few-shot cross-domain fault diagnosis, effectively accommodating distributional perturbations among different operating regimes. In comparison, the remaining methods, despite being serviceable in certain cases, appear less stable and less competitive overall.

**Figure 10:**
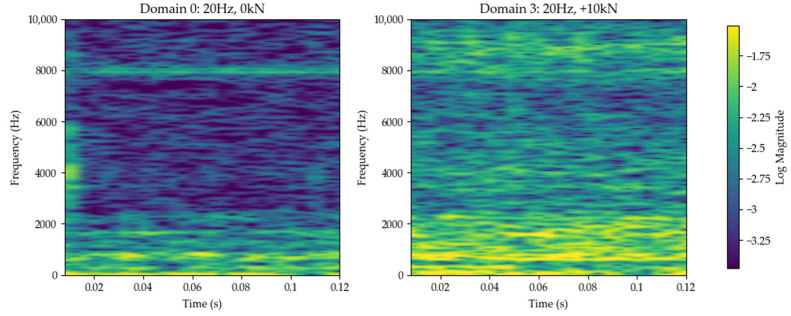
Time–frequency comparison between BJTU Domain 0 and Domain 3 under the same fault category.

Although WCBDC achieves the best average performance on the BJTU dataset, Table 5 and Table 6 show that several tasks involving Domain 3, such as 0→3, 3→0, 1→3, and 2→3, still obtain relatively lower accuracies. Since Domain 0 and Domain 3 have the same rotational speed but different lateral loads, Figure 10 compares their time–frequency distributions under the same fault category and signal length. The two domains show visible differences in energy distribution, suggesting that the +10 kN lateral load may alter fault-related vibration patterns and make feature alignment more difficult. In addition, tasks such as 1→3 and 2→3 involve simultaneous changes in speed and load, further increasing the domain discrepancy, especially in the 1-shot setting where each prototype is estimated from only one support sample.

#### 4.3.2. Ablation Experiments

The ablation results on the PU dataset, obtained under the 5-way 5-shot and 5-way 1-shot regimes, are respectively itemized in Table 7 and Table 8. This analysis is intended to isolate the incremental utility of the principal components, namely BDC and WC, within the proposed architecture. Four configurations are juxtaposed: the rudimentary baseline deprived of both BDC and WC, the BDC-equipped variant, the WC-equipped variant, and the integral WCBDC model.

On the PU dataset, the rudimentary baseline reaches an average accuracy of 82.03% for 5-way 5-shot tasks; after the insertion of BDC alone, this value ascends to 90.08%, while the isolated introduction of WC raises it to 86.70%. In the more austere 5-way 1-shot regime, the baseline attains 67.45%, which is subsequently lifted to 81.14% with BDC and 73.90% with WC. The full WCBDC construct invariably delivers the highest accuracy throughout all cross-domain settings, with mean accuracies of 92.19% and 84.13% in the 5-way 5-shot and 5-way 1-shot regimes, respectively. These empirical patterns indicate that BDC and WC each exert a positive effect on enhancing generalization and robustness for few-shot cross-domain fault diagnosis.

Table 9 and Table 10 summarize the corresponding ablation experiments on the BJTU dataset. For 5-way 5-shot tasks, the baseline achieves an average accuracy of 64.60%, improving to 71.14% with BDC and 71.01% with WC. An accuracy of 46.08% is achieved by the baseline for 5-way 1-shot tasks, which rises to 56.35% and 51.05% with BDC and WC, respectively. Under both settings, the full WCBDC model still gives the strongest results, averaging 77.24% in 5-way 5-shot diagnosis and 62.57% in 5-way 1-shot diagnosis. This further confirms that the embedding of BDC and WC effectively strengthens the cross-domain generalization capability and stability of the model under few-shot scenarios.

**Figure 11:**
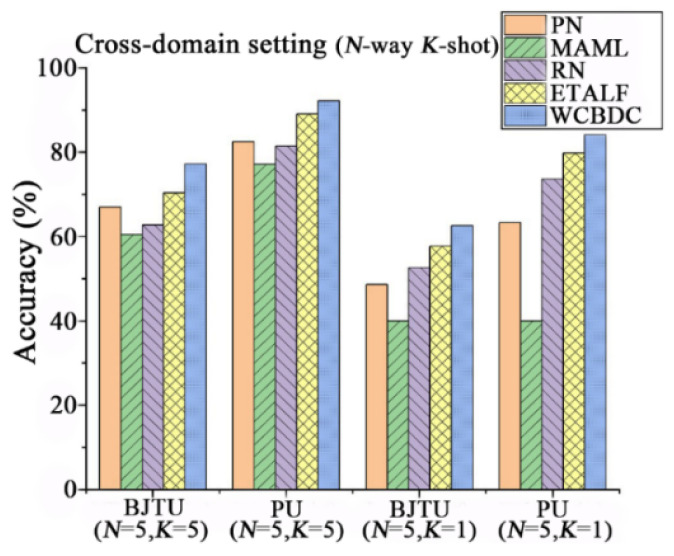
Average accuracies of comparative models across all the tasks.

**Figure 12:**
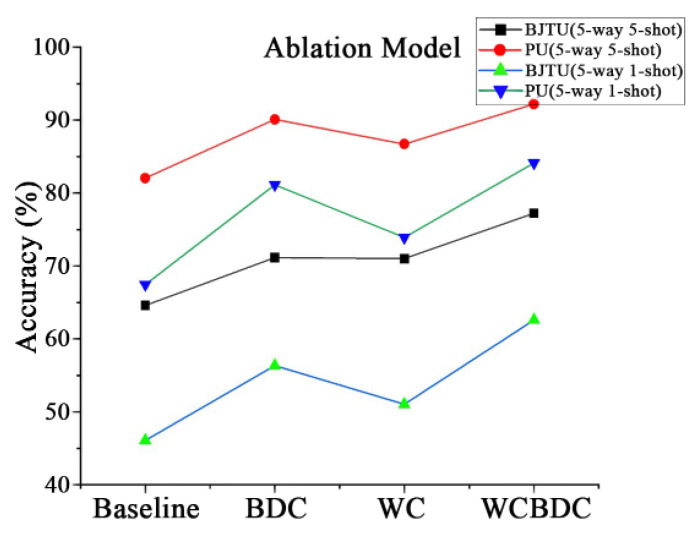
Task-aggregated mean accuracies of the ablation configurations.

Depicted in Figure 11 are the averaged accuracies of the competing architectures across the full suite of tasks. It is evident from the results that the proposed WCBDC construct invariably attains the preeminent accuracy under every tested scenario. Figure 12 illustrates the average accuracies from the ablation experiments. Systematic analysis of these results reveals the contributions of BDC and WC modules to model performance. Under the 5-way 5-shot setting, BDC and WC modules individually improve the accuracy by 6.54% and 6.41%, respectively, with no statistically significant difference observed between them, suggesting that both components provide comparable performance gains when sufficient support samples are available.

#### 4.3.3. Visualization and Analysis

In the more challenging 5-way 1-shot scenario, the performance gain of BDC (10.27%) significantly exceeds that of WC (4.97%). This aligns with the central limit theorem expectation: as the number of support samples decreases, feature distribution variance increases, and the correlation-based BDC module demonstrates stronger domain-shift mitigation. The complete WCBDC model achieves the best performance in all settings, with a synergistic gain of 6.22% in the 1-shot scenario, surpassing the individual contributions of each module. This synergy can be attributed to their complementary roles: BDC constructs embeddings with domain-shift suppression, while WC provides additional frequency-domain information, enhancing robustness under extremely limited samples.

**Figure 13:**
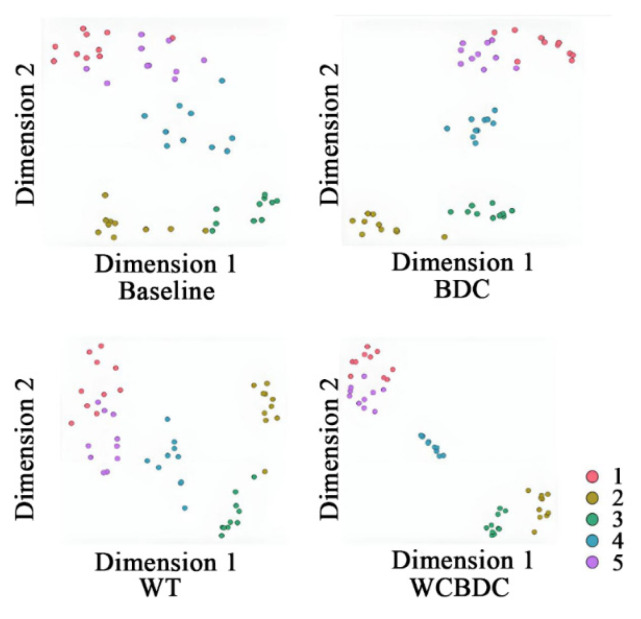
Embedding visualization of ablation models on the PU dataset.

**Figure 14:**
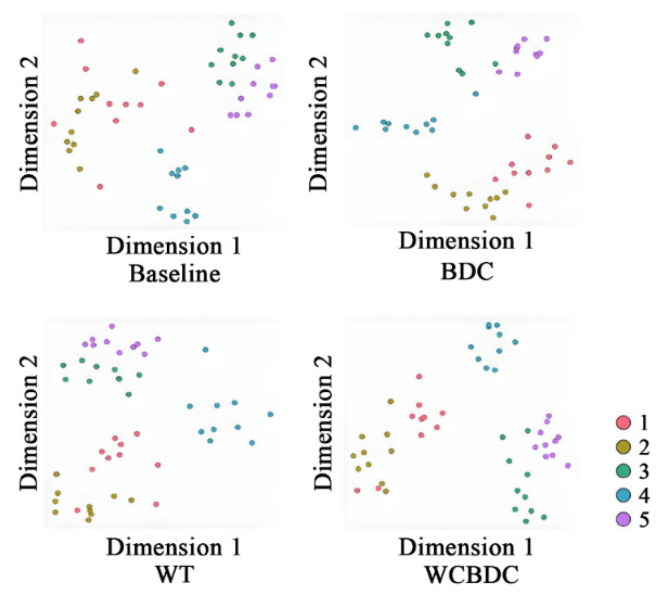
Embedding visualization of ablation models on the BJTU dataset.

For both the PU and BJTU datasets, t-distributed stochastic neighbor embedding (t-SNE) is adopted to project cross-domain feature embeddings into a visualizable space. Figure 13 and Figure 14 present the resulting representations of different ablation settings in a 5-way 5-shot meta-task, where each class encompasses 10 instances. Introducing BDC or WC to the baseline improves both inter-class separation and intra-class compactness. The combination of BDC and WC achieves the best separation and aggregation of embeddings, indicating that the proposed modules effectively alleviate feature distribution shifts across domains. However, the clustering of the same-class samples is still not optimal, suggesting potential areas for further improvement to enhance model effectiveness.

## 5. Conclusions

A metric-based meta-learning framework, referred to as WCBDC, is proposed in this paper for bearing fault diagnosis under small-sample and variable conditions. By incorporating the WC and BDC modules into a metric-oriented meta-learning paradigm, the method constructs a more discriminative and robust representation space for fault features. The key conclusions are delineated as follows:

1. The WC module extracts multi-scale time–frequency representations from raw vibration signals, enabling the capture of fault-related patterns across different frequency bands, and thereby enhancing the model’s adaptability under varying working conditions;
2. The BDC module improves feature discriminability from a statistical modeling perspective by characterizing inter-channel dependencies, which effectively increases inter-class separation while simultaneously reducing intra-class dispersion, leading to a more compact and well-structured feature distribution;
3. By jointly incorporating WC and BDC into the feature extraction stage of a metric-based meta-learning framework, WCBDC generally exhibits better performance in classification accuracy, cross-domain generalization capability, and diagnostic stability on two bearing datasets, in comparison with state-of-the-art approaches.

Nevertheless, the proposed method still has several limitations. First, the performance degradation in some PU transfer tasks, especially from high-speed domains to the low-speed domain, indicates that WCBDC is still affected by speed-induced frequency shifts. Second, the lower accuracies in several BJTU tasks suggest that severe lateral-load changes and compound speed–load shifts can introduce stronger domain discrepancies and more complex fault patterns. Third, although BDC improves feature discriminability, its pairwise channel-distance modeling and matrix operations may increase computational and memory costs as the channel dimension grows. Future work will focus on reducing speed-induced frequency shifts through speed normalization or order tracking, improving robustness to load and compound shifts through domain-alignment strategies, and developing lightweight BDC representations for practical industrial applications.

## Figures and Tables

**Table 1:** Operating-condition configurations for the PU bearing dataset.

Domain	RS (rpm)	Torque [Nm]	F_r [N]	Label
0	900	0.7	1000	N09_M07_F10
1	1500	0.1	1000	N15_M01_F10
2	1500	0.7	400	N15_M07_F04
3	1500	0.7	1000	N15_M07_F10

**Table 2:** Operating conditions of BJTU bearing dataset.

Domain	Motor Speed/Lateral Load
0	20 Hz/0 kN
1	40 Hz/0 kN
2	60 Hz/0 kN
3	20 Hz/+10 kN

**Table 3:** Comparison results on PU dataset, 5-way 5-shot accuracy (%).

Task	PN	MAML	RN	ETALF	WCBDC
0→1	79.79	66.37	77.33	90.22	**93.54**
0→2	82.70	69.20	80.75	89.61	**93.77**
0→3	82.69	70.99	75.44	87.82	**92.02**
1→0	78.16	74.28	73.62	84.62	**86.03**
1→2	87.51	82.80	91.61	94.84	**97.17**
1→3	84.89	81.30	86.25	91.55	**94.54**
2→0	74.74	75.64	71.51	82.06	**83.63**
2→1	87.53	84.17	87.92	92.61	**96.22**
2→3	85.68	83.46	88.17	92.42	**94.91**
3→0	75.51	73.47	71.92	80.98	**83.57**
3→1	84.83	79.97	85.38	90.46	**95.01**
3→2	85.62	81.07	87.44	91.77	**95.93**
Ave	82.47	76.89	81.45	89.08	**92.19**

**Table 4:** Comparison results on PU dataset, 5-way 1-shot accuracy (%).

Task	PN	MAML	RN	ETALF	WCBDC
0→1	60.22	46.13	70.41	77.80	**82.40**
0→2	62.67	46.50	69.69	79.98	**83.96**
0→3	64.12	30.44	67.44	78.42	**80.59**
1→0	56.06	35.64	66.38	73.41	**76.36**
1→2	70.87	36.11	82.80	88.08	**93.22**
1→3	67.46	26.79	80.03	84.15	**87.63**
2→0	54.35	47.35	63.41	69.70	**73.86**
2→1	66.94	48.37	79.20	86.21	**91.37**
2→3	67.25	34.20	78.99	84.90	**87.84**
3→0	57.88	41.67	66.22	67.84	**74.06**
3→1	64.50	44.26	78.23	81.94	**88.31**
3→2	67.02	41.96	80.49	84.95	**89.91**
Ave	63.28	39.95	73.61	79.78	**84.13**

**Table 5:** Comparison results on BJTU dataset, 5-way 5-shot accuracy (%).

Task	PN	MAML	RN	ETALF	WCBDC
0→1	77.54	67.35	70.55	78.50	**84.67**
0→2	76.27	67.74	73.47	79.38	**87.10**
0→3	56.35	46.38	47.13	57.90	**69.25**
1→0	77.18	56.13	77.65	76.95	**82.82**
1→2	77.60	64.71	72.05	78.26	**83.76**
1→3	54.46	33.55	51.41	63.03	**67.78**
2→0	68.89	60.37	70.56	74.46	**81.25**
2→1	72.91	57.25	68.93	73.95	**82.55**
2→3	57.10	30.56	42.60	52.37	**71.19**
3→0	61.45	32.54	62.40	69.35	**68.03**
3→1	58.51	35.35	57.13	68.26	**73.74**
3→2	65.29	30.46	59.60	71.63	**74.76**
Ave	66.96	48.53	62.79	70.33	**77.24**

**Table 6:** Comparison results on BJTU dataset, 5-way 1-shot accuracy (%).

Task	PN	MAML	RN	ETALF	WCBDC
0→1	59.99	46.13	58.86	67.91	**72.39**
0→2	58.91	46.50	65.12	68.93	**75.05**
0→3	38.63	30.44	38.95	45.55	**52.77**
1→0	55.59	35.64	65.44	63.58	**66.81**
1→2	55.15	36.11	63.63	66.58	**70.36**
1→3	35.59	26.79	40.23	43.48	**48.79**
2→0	52.39	47.35	61.06	62.40	**65.54**
2→1	56.35	48.37	57.15	63.75	**70.92**
2→3	37.43	34.20	33.77	41.04	**52.65**
3→0	40.74	41.67	48.68	**54.58**	52.79
3→1	42.46	44.26	45.69	55.58	**60.25**
3→2	50.45	41.96	52.27	58.91	**62.55**
Ave	48.64	39.95	52.57	57.72	**62.57**

**Table 7:** Ablation results on PU dataset, 5-way 5-shot accuracy (%).

Task	Baseline	BDC	WC	WCBDC
0→1	79.94	89.54	87.12	**93.54**
0→2	82.24	91.58	88.08	**93.77**
0→3	82.90	90.00	85.11	**92.02**
1→0	76.35	85.28	79.02	**86.03**
1→2	87.97	94.26	94.82	**97.17**
1→3	85.58	92.99	90.03	**94.54**
2→0	74.63	83.29	76.79	**83.63**
2→1	86.37	93.16	93.69	**96.22**
2→3	85.62	93.22	89.57	**94.91**
3→0	75.23	82.63	76.10	**83.57**
3→1	82.81	91.77	89.74	**95.01**
3→2	84.75	93.27	90.35	**95.93**
Ave	82.03	90.08	86.70	**92.19**

**Table 8:** Ablation results on PU dataset, 5-way 1-shot accuracy (%).

Task	Baseline	BDC	WC	WCBDC
0→1	63.55	78.83	70.38	**82.40**
0→2	64.37	80.46	71.29	**83.96**
0→3	65.36	78.98	69.97	**80.59**
1→0	60.94	76.24	63.38	**76.36**
1→2	74.91	89.40	85.18	**93.22**
1→3	72.87	85.59	79.45	**87.63**
2→0	59.48	71.42	63.06	**73.86**
2→1	73.11	86.64	84.26	**91.37**
2→3	73.80	85.91	79.26	**87.84**
3→0	60.18	70.53	63.01	**74.06**
3→1	69.98	83.43	78.18	**88.31**
3→2	70.81	86.26	79.34	**89.91**
Ave	67.45	81.14	73.90	**84.13**

**Table 9:** Ablation results on BJTU dataset, 5-way 5-shot accuracy (%).

Task	Baseline	BDC	WC	WCBDC
0→1	76.75	81.45	81.81	**84.67**
0→2	79.48	84.43	81.47	**87.1** **0**
0→3	60.14	64.75	61.66	**69.25**
1→0	74.25	77.48	78.75	**82.82**
1→2	73.99	78.36	77.56	**83.76**
1→3	53.39	61.12	58.31	**67.78**
2→0	68.18	74.53	75.49	**81.25**
2→1	73.99	78.16	76.55	**82.55**
2→3	54.85	60.15	59.15	**71.19**
3→0	50.55	63.42	62.75	**68.03**
3→1	51.54	62.95	69.18	**73.74**
3→2	58.11	66.96	69.52	**74.76**
Ave	64.60	71.14	71.01	**77.24**

**Table 10:** Ablation results on BJTU dataset, 5-way 1-shot accuracy (%).

Task	Baseline	BDC	WC	WCBDC
0→1	56.22	67.23	59.64	**72.39**
0→2	57.35	70.58	60.43	**75.05**
0→3	39.80	48.53	41.05	**52.77**
1→0	56.16	63.10	58.21	**66.81**
1→2	53.99	63.12	58.04	**70.36**
1→3	35.68	44.05	36.10	**48.79**
2→0	53.16	60.12	55.42	**65.54**
2→1	56.64	62.21	58.54	**70.92**
2→3	34.37	45.21	39.04	**52.65**
3→0	32.68	49.27	43.38	**52.79**
3→1	35.46	49.37	49.22	**60.25**
3→2	41.44	53.43	53.55	**62.55**
Ave	46.08	56.35	51.05	**62.57**

## Data Availability

The datasets used in this study are publicly available. The Paderborn University bearing dataset is available at https://mb.uni-paderborn.de/kat/forschung/bearing-datacenter/data-sets-and-download (accessed on 1 June 2026) and is described in [38]. The BJTU-RAO bogie dataset is available at https://drive.google.com/drive/folders/1RlZvFw-v07VvsL2Ni9cS7iFrTPDIhn2r?usp=sharing (accessed on 1 June 2026) and is described in [39].

## Abbreviations

The following abbreviations are used in this manuscript:

IFD	Intelligent fault diagnosis
STFT	Short-time Fourier transform
WT	Wavelet transform
CNN	Convolutional neural network
WCBDC	Wavelet convolution and Brownian distance covariance
WC	Wavelet convolution
BDC	Brownian distance covariance
PN	Prototypical network
MAML	Model-agnostic meta-learning
RN	Relation network
ETALF	Enhanced Transformer with Asymmetric Loss Function
LPF	Low-pass filter
HPF	High-pass filter
IWT	Inverse wavelet transform
GPU	Graphics processing unit
PU	Paderborn University
BJTU	Beijing Jiaotong University
RS	Rotational speed
t-SNE	t-distributed stochastic neighbor embedding
